# Potentiation of the Abscopal Effect by Modulated Electro-Hyperthermia in Locally Advanced Cervical Cancer Patients

**DOI:** 10.3389/fonc.2020.00376

**Published:** 2020-03-24

**Authors:** Carrie Anne Minnaar, Jeffrey Allan Kotzen, Olusegun Akinwale Ayeni, Mboyo-Di-Tamba Vangu, Ans Baeyens

**Affiliations:** ^1^Radiobiology, Department of Radiation Sciences, University of the Witwatersrand, Johannesburg, South Africa; ^2^Radiation Oncology, Wits Donald Gordon Medical Centre, Johannesburg, South Africa; ^3^Nuclear Medicine, Department of Radiation Sciences, University of the Witwatersrand, Johannesburg, South Africa; ^4^Radiobiology, Department of Human Structure and Repair, Ghent University, Ghent, Belgium

**Keywords:** modulated electro-hyperthermia, abscopal effect, radiotherapy, cervical cancer, immunomodulation

## Abstract

**Background:** A Phase III randomized controlled trial investigating the addition of modulated electro-hyperthermia (mEHT) to chemoradiotherapy for locally advanced cervical cancer patients is being conducted in South Africa (Human Research Ethics Committee approval: M1704133; ClincialTrials.gov ID: NCT03332069). Two hundred and ten participants were randomized and 202 participants were eligible for six month local disease control evaluation. Screening ^18^F-FDG PET/CT scans were conducted and repeated at six months post-treatment. Significant improvement in local control was reported in the mEHT group and complete metabolic resolution (CMR) of extra-pelvic disease was noted in some participants. We report on an analysis of the participants with CMR of disease inside and outside the radiation field.

**Method:** Participants were included in this analysis if nodes outside the treatment field (FDG-uptake SUV>2.5) were visualized on pre-treatment scans and if participants were evaluated by ^18^F-FDG PET/CT scans at six months post-treatment.

**Results:** One hundred and eight participants (mEHT: HIV-positive *n* = 25, HIV-negative *n* = 29; Control Group: HIV-positive *n* = 26, HIV-negative *n* = 28) were eligible for analysis. There was a higher CMR of all disease inside and outside the radiation field in the mEHT Group: *n* = 13 [24.1%] than the control group: *n* = 3 [5.6%] (Chi squared, Fisher's exact: *p* = 0.013) with no significant difference in the extra-pelvic response to treatment between the HIV-positive and -negative participants of each group.

**Conclusion:** The CMR of disease outside the radiation field at six months post-treatment provides evidence of an abscopal effect which was significantly associated with the addition of mEHT to treatment protocols. This finding is important as the combined synergistic use of radiotherapy with mEHT could broaden the scope of radiotherapy to include systemic disease.

## Introduction

The abscopal effect is a systemic response to ionizing radiation (IR) in which non-irradiated lesions respond after irradiation of the primary treatment site (1, 2). It is generally accepted that the abscopal effect is driven by underlying immune mechanisms which are activated by IR (2–4). One proposed mechanism is the immunogenic cell death (ICD) caused by IR (3) which requires the release of damage associated molecular patterns (DAMPs). These in turn activate dendritic cells and enhance antigen expression and presentation to the immune system. Ionizing radiation has also been shown to enhance the functioning of T-cells (4).

The frequency of reported abscopal effects in the literature is extremely low with only a handful of published cases per year (3, 4). In a review, Reynders et al. summarized 23 case reports, one retrospective study, and 13 pre-clinical papers, from the 1970s to 2014. Only one of these involved a primary squamous cell carcinoma of the cervix. The patient (age 69 years) was treated with external beam radiation (EBRT) and brachytherapy (BT) for locally advanced cervical cancer (LACC) and showed a complete response of the para-aortic nodes outside of the radiation field, as well as a complete response of the tumor, on the post-treatment Abdominal and Pelvic Computed Tomography (CT) and Pelvic Magnetic Resonance Imaging (MRI) scans (5). Reynders et al. concluded that the abscopal effect is based on anti-tumor immunity and was more common in immunogenic tumor types. Renal cell carcinoma had the most frequently reported cases of the abscopal effect followed by hepatocellular carcinoma. The abscopal effect was observed at all ages and with a variety of radiotherapy protocols. The preclinical data indicates that some immunomodulatory agents may have potential to act synergistically with IR to induce a systemic response (4) which may explain the increase in the number of reported abscopal effects with the combined treatment of immunotherapies and IR (6).

The addition of mild hyperthermia to local irradiation has shown to have immunomodulating effects which may result in enhanced tumor regression and an abscopal effect when combined with radiotherapy, as was seen in a liposarcoma patient treated with hyperthermia and radiotherapy (7). Hyperthermia may directly activate the immune cells present in the tumor and its microenvironment (8) and may further enhance the function of the dendritic cells (9).

Modulated electro-hyperthermia (mEHT) applies amplitude modulated radiofrequency (13.56 MHz), in a capacitive coupling set-up to target and heat malignant tissues, sensitizing them to treatments. The technique exploits the differences in impedance between the malignant and healthy tissue as well as impedance matching technology, to selectively deliver an energy to the malignant tissues. The energy deposition has the net effect of an increase in the thermal energy, and temperature. The biophysics are further described in detail in the literature (10–12). Preclinical research suggests that mEHT combined with immunotherapies is able to elicit an immune-mediated response (13) which may even extend to untreated tumors. Vancsik et al. showed that mEHT induced DAMPs in murine models was followed by an invasion of antigen presenting cells (APC) and T-cells at the site of the treated tumor and that when mEHT was administered combined with a T-cell stimulating agent, APC and T-cell invasion was also seen in the untreated tumors of the same murine model (14). In an *in vivo* study, mEHT combined with dendritic cell therapy elicited a response to untreated tumors in murine squamous cell carcinoma (SCCVII) models (15). Ionizing radiation has shown to increase the expression of immunogenic molecules such as calreticulin, on the surface of tumor cells and radiation-induced stress-response leads to the expression of heat shock protein70 (HSP70) on cell membranes. This Heat Shock Protein plays an important role in mounting an immune response at the site when released into the extracellular matrix (16). Yang et al. reported an increased release of the expressed HSP70 and increased levels of calreticulin after mEHT, compared to other heating methods (17).

The safety and heating efficacy of mEHT in cervical cancer patients has been demonstrated (18–20). Minnaar et al. (19) reported on local disease control in an ongoing randomized controlled trial investigating the effects of the addition of mEHT to chemoradiotherapy (CRT) protocols for the treatment of LACC. The trial was conducted in a resource-constrained setting and in high risk patients in South Africa. In the report, 202 participants were eligible for six month local disease-free survival (LDFS) and local disease control (LDC) (mEHT: *n* = 101; Control: *n* = 101), of which 171 [mEHT: *n* = 88 (87.1%); Control: *n* = 83 (82.2%)], were alive at six months post-treatment. Participants in the mEHT group had a higher LDC and complete metabolic response of the tumor (45% and 58%), than those in the Control Group (24% and 36%), (*p* = 0.005 and *p* = 0.003, respectively), and were significantly more likely to achieve six month LDFS (OR: 0.36, 95% CI: 0.19-0.69; *p* = 0.002) (19). During the LDC analysis, it was noted that some of the participants with extra-pelvic disease present on the pre-treatment Fluorodeoxyglucose (^18^F-FDG) Positron Emission Tomography (PET) /CT scans showed a complete metabolic resolution (CMR) of disease outside the treatment field on the post-treatment ^18^F-FDG PET/CT scans. An analysis of the subset of patients with extra-pelvic disease visualized on the pre-treatment ^18^F-FDG PET/CT scans was subsequently planned. We present the results of this analysis with the aim of investigating the possibility of an abscopal effect induced by the addition of mEHT to CRT in these participants.

## Methods and Materials

A Randomized controlled trial by Minnaar et al. (19) is being conducted at the Charlotte Maxeke Johannesburg Academic Hospital, a public hospital in Johannesburg, South Africa, by the Radiation Sciences department of the University of the Witwatersrand. The trial was registered on the South African National Clinical Trials Register before recruitment was started (ID:3012) and approval from the Human Research Ethics Committee was obtained (M704133/M190295). The trial was registered at ClincialTrials.gov (NCT03332069). Enrolment began in January 2014 and was closed in November 2017.

Two hundred and ten participants were randomized to receive either CRT alone (Control Group) or combined with mEHT (mEHT Group). Randomization was conducted using the REDCap on-line computer generated random-sampling tool with stratification according to HIV status and accounting for age and FIGO stage. Physicians reporting on the ^18^F-FDG PET/CT scans were blinded to treatment allocation and did not interact with the participants, eliminating the risk of biased reporting.

### Eligibility

Eligibility criteria for the trial: Females with International Federation of Gynecology and Obstetrics (FIGO) (21) stages IIB to IIIB primary, treatment naïve, histologically confirmed squamous cell carcinoma of the cervix (staged based on clinical examination, chest radiography, and a pelvic ultrasound) eligible for CRT with radical intent; Signed informed consent; >18 years old; Eastern Cooperative Oncology Group (ECOG) score <2; Creatinine clearance >60 mL/min. Screening evaluations included full blood count, urea and creatinine levels, liver function, Human Immunodeficiency Virus (HIV) test; and a CD4 count if necessary. An ^18^F-FDG PET/CT scan was performed on eligible participants prior to commencement of therapy, as a baseline study against which response to treatment could be measured. Participants with bilateral hydronephrosis, visceral metastases, or fistulas visualized on the ^18^F-FDG PET/CT scan were excluded from the study. HIV-positive patients were included provided their CD4 count was above 200 cells/μL and/or they had been on antiretroviral therapy (ART) for more than six months.

Exclusion Criteria for the trial: Bilateral hydronephrosis; Second primary malignancy/prior malignancy treated in the preceding two years; vesicovaginal fistula or rectovaginal fistula that required a change in treatment protocols; Abnormal liver function tests; Pregnant or breast feeding; Prior hysterectomy; Cardiovascular disease (excluding controlled hypertension); Acute or life-threatening infections or medical conditions; Contraindications to any of the prescribed treatments.

At the time of this analysis all participants were a minimum of six months post-treatment and local disease control data at six months post-treatment was available for all participants (19). Participants were considered eligible for the sub-analysis presented in this report if: They met all the trial eligibility criteria; the pre-treatment ^18^F-FDG PET/CT scan showed FDG-avid (SUV > 2.5) nodal disease outside of the pelvic treatment field; and the participants had a post-treatment ^18^F-FDG PET/CT scan.

### Data Management

Participant data was captured using REDCap (Research Electronic Data Capture), an online, secure web based application hosted by the University of the Witwatersrand.

### Treatment

All participants were planned to receive 50Gy in 25 fractions EBRT to the whole pelvis and 24Gy in 3 fractions of high dose rate (HDR) BT (36Gy equivalent dose in 2Gy fractions for an alpha-beta ratio of 10; source used: Iridium-192) and two doses of cisplatin (80 mg/m2) administered 21 days apart (subject to the participant's fitness to receive cisplatin), as per institutional protocol. The goal of RT was for participants to receive a total dose of 86Gy equivalent by the combination of EBRT and BT. External beam radiation to the whole pelvis was delivered using a two dimensional four-field-box technique to include the tumor and pelvic nodes. Participants were simulated supine. The superior border of the Anterior-Posterior and Posterior-Anterior (AP-PA) field was mid-L5. The inferior border was either the inferior part of the ischial tuberosity or the lowest extension of the tumor with at least a 2 cm margin, whichever was lower. The lateral borders were 2 cm beyond the lateral margins of the bony pelvis. For the lateral fields, the superior and inferior borders were the same as for the AP-PA fields. The anterior border was the mid to anterior third of the symphysis pubis and the posterior border was S2–S3 to include the presacral nodes and possible tumor extension along the uterosacral ligament.

Modulate electro-hyperthermia (Model: EHY2000+; Manufacturer: Oncotherm GmbH, Troisdorf, Germany) was administered twice per week (maximum ten treatments), at a maximum power of 130 W, immediately before EBRT (maximum 30 min from completion of mEHT to completion of EBRT). Step-up heating protocols were adhered to and mEHT treatments were administered at least 48 h apart. A 30 cm diameter round electrode was used and treatment duration was 55 min at the final power output, with a minimum planned energy dose of 360 KJs. Details of the technique are described elsewhere in the literature (11, 19, 22).

### Outcome Measures

Nodes with FDG-avid disease were grouped by region on the pre-treatment scans: Head and Neck; Thorax; Abdomen (including the upper pelvis outside of the radiation field); and Pelvis (within the radiation field). The standard uptake value (SUV) cut-off was considered to be 2.5 and evaluation of the ^18^F-FDG PET/CT scans was based on PERSIST 1.0 Criteria. Tumor response was classified as Complete Metabolic Response (CMR); Partial Metabolic Response (PMR); Stable Metabolic Disease (SMD); Progressive Metabolic Disease (PMD) (23). On the follow-up scans each region was scored as: no change; resolved nodes; new nodes. Only the complete metabolic response of all disease (nodes outside of the radiation field, nodes inside the radiation field, and the tumor), as visualized on post-treatment ^18^F-FDG PET/CT scans, was considered an indicator of the abscopal effect.

### Statistics

The frequency of the observed abscopal effect was compared by group (mEHT or Control) and HIV status (positive or negative) using a Chi-squared frequency table. Paired *t*-test was used to compare the difference in means between groups and logistic regression was used to test prognostic factors. Two sided *p* values are reported and *p* < 0.05 were considered significant. STATA 13.0 Statistics software program (Stata Corporation, College Station, Texas, USA), was used to analyze the data.

### Ethics

All procedures performed in studies involving human participants were in accordance with the ethical standards of the institutional and/or national research committee (M120477 and M190295) and with the 1964 Helsinki declaration and its later amendments or comparable ethical standards.

## Results

### Characteristics

**Figure 1:**
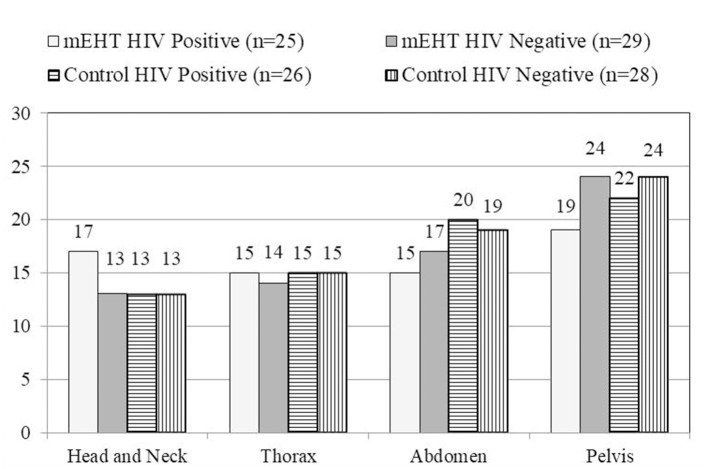
Number of patients with nodes visualized by region. The number of participants with nodes visualized in each region is represented graphically, showing a similar pattern in all participants in each treatment group on the pretreatment ^18^F-FDG PET/CT. mEHT, Modulated Electro-Hyperthermia; HIV, Human Immunodeficiency Virus.

Two hundred and ten participants were randomized for treatment, of which 146 [70%] had FDG-avid nodal disease visualized outside of the radiation field on the pre-treatment ^18^F-FDG PET/CT scans (mEHT Group: *n* = 68 [64%]; Control Group: *n* = 78 [75%]). One hundred and eight of the participants with extra pelvic nodal disease survived six months post-treatment and were eligible for the post-treatment ^18^F-FDG PET/CT scans (mEHT Group: *n* = 54 [79%]; Control Group: *n* = 54 [69%]) and were therefore included in this analysis. The characteristics, including treatment characteristics, of these 108 participants are listed in Table 1. The number of participants (grouped by treatment group and HIV status) with nodes visualized in each region on the pre-treatment ^18^F-FDG PET/CT scans are shown Figure 1. The median number of weeks between the final RT treatment and the follow-up ^18^F-FDG PET/CT scans was 26.3 in the mEHT Group (Q1: 25.3; Q3: 27.3) and 27 in the Control Group (Q1: 26; Q3: 29).

**Table 1:** Characteristics of participants eligible for analysis of the abscopal effect.

		**mEHT**	**Control**	
		**54**	**54**	
FIGO Staging	IIB	25 [46%]	22 [41%]	
	III	29 [54%]	32 [59%]	
Race	African	51 [94%]	52 [96%]	
	Caucasian	1 [2%]	0 [0%]	
	Other	2 [4%]	2 [4%]	
Age [years]	Mean	49.3	49.9	*p* = 0.776
	SD	9.98	9.99	
	Range	30–68	28–70	
BMI	Mean	28.7	27.0	*p* = 0.127
	SD	5.61	6.01	
	Range	18–44	15–39	
Total RT dose (EQD2)	Mean	85.7Gy	86Gy	*p* = 0.251
	SD	1.65	0	
	Range	74–86Gy	86Gy	
No of Cisplatin doses	Mean	1.37	1.25	*p* = 0.321
	SD	0.69	0.76	
	0 doses	5 [9%]	6 [11%]	
	1 dose	19 [35%]	20 [37%]	
	2 doses	30 [56%]	28 [52%]	
Days between final RT and PET/CT	Mean	188.2	193.4	*p* = 0.242
	SD	24.05	22.15	
	Range	54–310	155–266	
CD4 count [cells/μL]	Mean	552.9	543.9	*p* = 0.089
	SD	264.15	276.36	
	Range	194–1077	134–1524	
No of mEHT doses	Mean	9.54		
	SD	1.07		
	Range	4–10		
Average KJ administered during mEHT	Mean	382.6 KJ		
	SD	29.95		
	Range	259–427 KJ		

*No significant differences in characteristics and treatment were seen between the two groups. mEHT, Modulated Electro-Hyperthermia; FIGO, Federation of Gynecology and Obstetrics; SD, Standard Deviation; BMI, Body Mass Index; RT, Radiation Therapy; PET/CT, Positron Emission Tomography / Computed Tomography; KJ, Kilojoules*.

### Abscopal Effect as Visualized on ^18^F-FDG PET/CT

**Figure 2:**
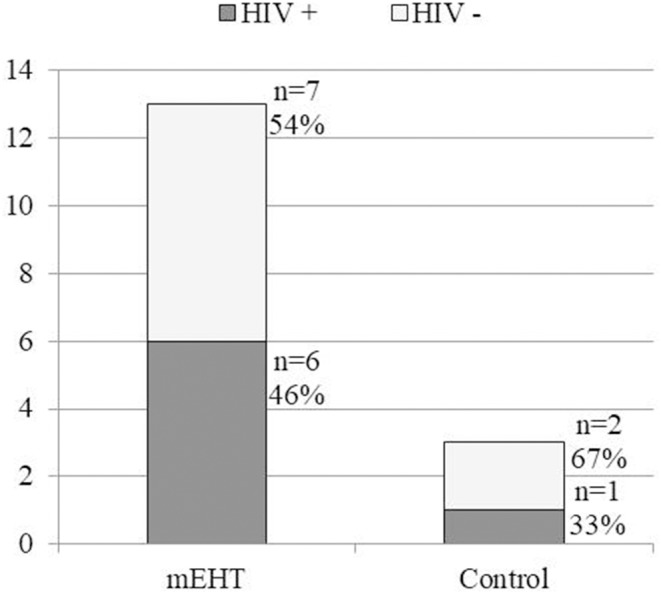
Frequency of observed abscopal effect in HIV-positive and HIV-negative participants in each treatment group. A significant difference between the frequency of abscopal effect was noted between the mEHT Group (13 out of 54[24.1%]) and the Control Group (3 out of 54 [5.6%]) (*p* = 0.013). There was no significant difference in frequency of the observed abscopal between the HIV-positive and HIV-negative participants. mEHT, Modulated Electro-Hyperthermia; HIV, Human Immunodeficiency Virus.

An abscopal response was only considered if all disease, including the primary tumor, nodes within the radiation field, and all nodes outside of the radiation field showed a complete metabolic response (SUV <2.5) on the six month post-treatment ^18^F-FDG PET/CT. Therefore all participants who had an abscopal effect also showed local disease control (a complete metabolic response of the tumor and nodes within the pelvic radiation field). The percentage of participants with complete resolution of all metabolically active disease on six month post-treatment ^18^F-FDG PET/CT scans was higher in the mEHT group: *n* = 13 [24.1%] than in the control group: *n* = 3 [5.6%] (Chi-squared: *p* = 0.013). There was no significant difference in the response between the HIV-positive (*n* = 51) and -negative (*n* = 57) groups (HIV-positive: *n* = 7 [13%]; HIV-negative: *n* = 9 [16%]; Chi-squared: *p* = 0.793) with a close to even split in frequency of abscopal responses observed between the HIV-positive and -negative participants in each treatment group, as seen in Figure 2. In a multivariate analysis (confidence interval [CI] 95%) of age, cisplatin cycles, total radiation dose, and the number of days between the final radiation treatment and the follow-up ^18^F-FDG PET/CT, none of the variables were indicators of an abscopal effect (Age: OR:1.01, *p* = 0.692, CI: 0.96-1.07; Cisplatin cycles: OR: 1.20; *p* = 0.671; CI: 0.51-2.83; Days to PET/CT: OR: 1.01; *p* = 0.283; CI: 0.99-1.07; Total RT: OR: 0.66; *p* = 0.316; CI: 0.30-1.47). In a univariate analysis, the CD4 count of participants was also not predictive of an abscopal effect (OR: 1.00, *p* = 0.893, CI: 0.997–1.003). In the participants in whom an abscopal effect was observed, the mean time between the final radiation and the follow-up ^18^F-FDG PET/CT was 196 days (range 162–266).

### Follow up

One participant had visceral disease on the pre-treatment ^18^F-FDG PET/CT scan: multiple lung nodules (highest SUV in the left lung of 6.03 and the right lung of 4.38) and a lesion in the T11 vertebra (SUV 9.71). This participant was in the mEHT group. The follow-up ^18^F-FDG PET/CT scan showed no sign of metabolically active disease. This participant is 18 months post-treatment and is still disease free. Of the participants who showed an abscopal effect, seven out of 13 mEHT participants and two out of three control participants have reached 2 years post treatment and are still disease free. Two participants in the mEHT group and one in the Control group demised before reaching two years (cause of death: acute renal failure). Four participants in the mEHT group have not yet reached two years post treatment (two are 18 months and two are 12 months post-treatment), however they are still disease free. Table 2 lists all the sites of FDG avid disease seen in the participants in whom an abscopal response was observed, the disease-free survival observed, the viral load, CD4 count, and the HIV status of the participants.

**Table 2:** Details of the extra-pelvic disease in participants with an ^*^Abscopal Effect.

**HIV status**	**Days to PET**	**No. ChT**	**Description of extra-pelvic disease on pre-treatment PET/CT**	**Survival**
**mEHT group**				
Pos. (CD4: 863; VL:27)	211	2	Common carotid (SUV 7.5); Para tracheal (SUV 4.41); Axillary (SUV 5.59)	2YDFS
Pos. (CD4:194; VL: <20)	163	1	Bilat. Jugular. digastric (SUV Left: 2.62; Right: 3.34); Axillary (SUV 4.49); Pre- (SUV 2.75) and Sub-carinal (SUV 3.16); Retrocrural (SUV 3.47) Bilat. PA (SUV Left: 2.59; Right: 3.39)	OS: 335
Pos. (CD4: 905; VL: <20)	200	2	Jug. Digastric (SUV 2.6); Hilar (SUV 2.92)	2YDFS
Pos. (CD4: 845; VL: ND)	190	2	Bilateral supraclav. (SUV Right: 6.04; Left: 2.94)	2YDFS
Pos. (CD4: 456; VL: ND)	192	2	7 Bilat. cervical (highest SUV4.81); Axillary (SUV 4.24); Subcarinal (SUV 2.73); CI (SUV Right: 2.93; Left: 4.73)	2YDFS
Pos. (CD4: 284; VL:196)	182	2	Bilat. Jug. Digastric (SUV Right: 7.42; Left: 2.72); CI (SUV 3.89)	DF at 18 m
Neg.	185	1	Aorto-pulmonary (SUV 2.75)	2YDFS
Neg.	197	1	PA (SUV 4.73)	2YDFS
Neg.	185	2	Cervical (Level IIA SUV 5.67; Level IIB SUV 3.49)	2YDFS
Neg.	183	0	Bilat. axillary (SUV Left: 2.87; Right 2.98)	OS: 596
Neg.	193	2	Supraclav. (SUV 7.42); Paratracheal (SUV 20.38); Aorto-pulmonary (SUV 14.72); Bilat. hilar / Peribronchial (SUV Right 17.86, Left 13.47); Multiple Pulmonary nodules (SUV Right 6.03, Left 4.38); T11 (SUV 9.71)	DF at 18 m
Neg.	224	1	Coelic axis (SUV 7.93)	DF at 12 m
Neg.	189	2	Aorto-pulmonary (SUV 4.1); Pre-carinal (SUV 3.7)	DF at 12 m
**Control**				
Pos. (CD4: 564; VL: ND)	207	0	PA (SUV 3.35); CI (SUV 4.5 0.9)	OS: 483
Neg,	266	2	Aorto-pulmonary (SUV 2.79); PA (SUV 4.21)	2YDFS
Neg.	183	2	PA (SUV 4.81); Paravertebral (SUV 6.56)	2YDFS

* *An abscopal response was considered if there was complete metabolic resolution of the extra-pelvic disease and pelvic disease, including the tumor and pelvic nodes, on the post-treatment ^18^F-FDG PET/CT scans, with an SUV of <2.5. ChT, Chemotherapy; CI, Common Iliac; DF, Disease Free; HIV, Human Immunodeficiency Virus; mEHT, Modulated Electro-Hyperthermia; ND, Not Detectable; OS, Overall Survival; PA, Para aortic; SUV, Standard Uptake Value; YDFS, Years of Disease Free Survival; VL, Viral Load*.

### HIV Status

In order to rule out the effects of HIV on the visualization of nodes, the cases were reviewed with the intention of discarding cases which had nodes known to be visualized in HIV disease. HIV-positive participants with high viral load levels may have benign hypermetabolic foci visualized on ^18^F-FDG PET/CT images, resulting in false positive interpretations of malignancy (24). Furthermore, Sathekge et al. showed that the CD4 count of HIV positive participants was inversely proportional to the FDG uptake in the nodes (25). During acute HIV infection FDG uptake increases in the head and neck lymph nodes, in mid stage of HIV infection hypermetabolism occurs in cervical, axillary, and inguinal lymph nodes, and an increased FDG uptake occurs in the colon, mesenteric, and ileocecal lymph nodes during late HIV disease (24). None of our participants were in acute (newly diagnosed) or late stage (no Acquired Immune Deficiency Syndrome-defining illnesses other than cervical carcinoma) of HIV infection. Four of the participants with an abscopal response showed increased FDG uptake in the axillary glands: one was HIV-negative and was therefore still included, three were HIV-positive and all had increased FDG uptake in extra-pelvic nodes other than the axillary nodes. These three participants were therefore still included. Of the seven HIV-positive participants, one had increased FDG uptake in the inguinal nodes however several other extra-pelvic nodes were also visualized and the patient was included.

## Discussion

The CMR of disease outside the radiation field at six months post-treatment in our sample provides evidence of an abscopal effect. The frequency of the observed abscopal effect was significantly associated with the addition of mEHT. This finding is important as methods to enhance the abscopal effect could broaden the scope of ionizing radiation from a local treatment to a systemic and potentially curative modality for metastatic and systemic disease. The abscopal effect was seen equally in HIV-positive and -negative participants in the group treated with mEHT. This suggests that the potentiation of the systemic, immune-mediated response to IR was not inhibited by HIV-infection and could still be possible in such high risk patients.

Reynders et al. reported that the median time to achieve an abscopal response was five months, ranging from 1 to 24 months (4). In our study we assessed the abscopal effect as part of the disease response at six months, which corresponds to the findings by Reynders et al. In their review Reynders et al. report on patients who had received multiple fractions of radiotherapy followed by a reduction in size/metabolic activity of a non-irradiated lesion (partial response). In our report we present only participants who showed a complete metabolic response of all disease, including the primary tumor. This strengthens the probability of an abscopal response in our participants. Reynders et al. excluded papers in which systemic cytotoxic drugs were administered (4). We have included participants who were treated with cisplatin as a radiosensitiser, however the administration of cisplatin to participants in the mEHT Group and Control group was evenly matched suggesting that the difference between responses in the two groups was not the due to the cisplatin and is associated with the addition of mEHT. Furthermore cisplatin was not a predictor of an abscopal effect in our sample.

The rarity of the abscopal effect documented in the literature suggests that the abscopal effect alone is unlikely to impact clinical regimes and influence treatment choices (3). Considering their immune components, the combination of radiotherapy with immunotherapies and mEHT may provide an opportunity to boost abscopal response rates. Reynders et al. reported on four case reports of the abscopal effect using Ipilimumab (one in adenocarcinoma of the lung and three in melanoma patients), one using BCG-vaccination (adenocarcinoma of the lung) combined with IR, and one retrospective study in which 11 out of 21 melanoma patients treated with Ipilimumab followed by palliative radiotherapy showed an abscopal response (4). The increase in use of immunotherapies combined with IR has resulted in an increase in the reports of abscopal effects. At least ten trials have been registered on ClincialTrials.gov to investigate the effects, including the abscopal effect, of immunotherapies combined with IR. The lack of reliable biomarkers to predict and confirm the presence of an abscopal effect may impact on the future optimization of protocols to induce an abscopal effect. An important future field of investigation is therefore the development of biomarkers which can reliably predict and quantify the presence of an abscopal effect.

Preclinical data on the synergistic effects of immunomodulating agents and mEHT with IR, as well as case reports, and the results of this study, provide strong support for the development of trials on the combined use of IR with mEHT and immunotherapies. Positive results in such trials would broaden the scope of ionizing radiation from local or palliative treatment to a potentially curative modality in metastatic and systemic disease.

## Data Availability Statement

The raw data supporting the conclusions of this article will be made available by the authors, without undue reservation, to any qualified researcher.

## Ethics Statement

All procedures performed in studies involving human participants were in accordance with the ethical standards of the institutional and/or national research committee (Human Research Ethics Committee approval number: M120477) and with the 1964 Helsinki declaration and its later amendments or comparable ethical standards. Informed consent was obtained from all individual participants included in the study.

## Author Contributions

CM: conducted the research, gathered the data, statistical analysis, interpretation of results, and writing of the manuscript. JK: planning and prescribing of treatments, oversaw the treatment and follow-ups of trial participants, and reviewed manuscript. OA: reviewed ^18^F-FDG PET/CT reports, reviewed manuscript. M-D-TV: oversaw all the ^18^F-FDG PET/CT scans and related logistics, reviewed manuscript. AB: supervised data collection and data quality control, project planning, management of funding, and reviewed manuscript.

### Conflict of Interest

The authors declare that the research was conducted in the absence of any commercial or financial relationships that could be construed as a potential conflict of interest.
